# Impact of HVT Vaccination on Splenic miRNA Expression in Marek’s Disease Virus Infections

**DOI:** 10.3390/genes10020115

**Published:** 2019-02-05

**Authors:** Julie A. Hicks, Hsiao-Ching Liu

**Affiliations:** Department of Animal Science, North Carolina State University, Raleigh, NC 27695, USA; jahicks3@ncsu.edu

**Keywords:** Marek’s disease virus, microRNA, transcriptional regulation

## Abstract

Marek’s Disease is a lymphoproliferative disease of chickens caused by Marek’s Disease Virus. Similar to other herpesviruses, Marek’s Disease Virus (MDV) encodes its own small non-coding regulatory RNAs termed microRNAs (miRNAs). We previously found that the expression profile of these viral miRNAs is affected by vaccination with Herpesvirus of Turkeys (HVT). To further characterize miRNA-mediated gene regulation in MDV infections, in the current study we examine the impact of HVT vaccination on cellular miRNA expression in MDV-infected specific-pathogen-free (SPF) chickens. We used small RNA-seq to identify 24 cellular miRNAs that exhibited altered splenic expression in MDV infected chickens (42 dpi) compared to age-matched uninfected birds. We then used Real Time-quantitative PCR (RT-qPCR) to develop expression profiles of a select group of these host miRNAs in chickens receiving the HVT vaccine and in vaccinated chickens subsequently infected with MDV. As was seen with viral miRNA, host miRNAs had unique splenic expression profiles between chickens infected with HVT, MDV, or co-infected birds. We also discovered a group of transcription factors, using a yeast one-hybrid screen, which regulates immune responses and cell growth pathways and also likely regulates the expression of these cellular miRNAs. Overall, this study suggests cellular miRNAs are likely a critical component of both protection from and progression of Marek’s Disease.

## 1. Introduction

Marek’s Disease (MD) is a lymphoproliferative disease in chickens. Marek’s Disease Virus (MDV), the causative agent of MD, is an alpha herpesvirus which serves as a classical model for virally-induced tumors. Commercial vaccines have been developed which can prevent disease progression. One of the most commonly used is Herpesvirus of Turkeys (HVT), a closely related alpha herpesvirus. Though it is unable to prevent infection, HVT-vaccinated birds are usually asymptomatic when subsequently infected with an oncogenic MDV strain.

As large double-stranded DNA viruses, herpesviruses encode many diverse genes, including small non-coding RNAs, termed microRNAs (miRNAs). MDV-encoded miRNAs have been shown to regulate a number of both cellular and viral processes [1]. MDV miRNAs are located in two major clusters in the viral genome, one cluster is adjacent to the *MEQ* oncogene and the other is located near ICP4, a member of the latency associated transcript (*LAT*) [2]. It has been suggested that MDV miRNAs are involved in virally-induced cellular transformation as many have higher expression in tumor versus non-transformed cells [3]. HVT also encodes its own miRNAs, which are mainly located within the long-repeat regions of the genome [4]. We have previously demonstrated that these virally-encoded miRNAs are dynamically expressed between chickens infected with MDV, HVT, or co-infected [5]. We found that five MDV-encoded miRNAs have differential splenic expression between MDV-infected birds and in those co-infected with HVT and MDV at 42 days post infection (dpi) [5]. Of the HVT-encoded miRNAs, six were differentially expressed between the spleens of birds solely vaccinated with HVT and those co-infected with HVT and MDV [5]. In silico target mapping suggested that these differentially expressed viral miRNAs likely regulate a number of cellular processes, including cell proliferation and the adaptive immune response [5].

Numerous studies have shown that in addition to encoding their own miRNAs, herpesviruses greatly impact the expression profile of host miRNAs. These alterations in cellular miRNA expression have been linked to dueling roles. In some instances, virally-induced changes in cellular miRNA expression are part of the host’s response to infection and are detrimental to the virus. In other instances, changes are induced by the virus itself to produce a more amiable environment for viral replication. The cellular protein mitochondrial ATP synthase subunit β (ATP5B) facilitates viral replication in Herpes Simplex Virus 1 (HSV1) infected cells [6]. The cellular miRNA *miR-101* was found to target *ATP5B* and increased *miR-101* expression leads to a reduction in HSV1 replication [6]. On the other hand, increased expression of *miR-23a* results in increased HSV1 replication [7]. This enhanced viral replication was linked to *miR-23a* suppression of interferon regulatory factor 1 (IRF1) expression [7]. Cells infected with Human Cytomegalovirus (HCMV) have reduced levels of *miR-21* [8]. This reduction in *miR-21* allows for an increase in the levels of its target CDC25A. Ectopic expression of *miR-21* in HCMV-infected cells reduced both viral gene expression and progeny production. Interestingly, three viral proteins, IE1, pp71, and UL26 are miR-21 transcriptional regulators [8]. 

Cellular miRNAs are also important mediators of the lytic/latent switch in herpesvirus infected cells. For example, the miRNAs *miR-200b* and *miR-429* regulate the expression of the cellular transcription factors ZEB1 and ZEB2 [9]. Reduction of ZEB1 and ZEB2 by *miR-200b* and *miR-429* during Epstein-Barr Virus (EBV) latency, induces the switch to lytic replication [9]. *MiR-200b* and other members of the *miR-200* family target the immediate early HCMV gene *UL122* [10]. Repression of *UL122* by these miRNAs is critical for latency maintenance, as an HCMV mutant lacking the *miR-200* family binding site abrogates the lytic/latent switch [10]. The well-known immunomodulatory miRNA, *miR-146a*, is greatly increased in EBV lytically-infected cells and leads to a reduction in viral production [11]. Overall, these studies demonstrate that cellular miRNAs play a myriad of important roles in the herpesvirus lifecycle.

The expression of cellular miRNAs is impacted by MDV infection. Cultured chicken embryonic fibroblasts (CEFs) infected with MDV exhibit higher expression of the cellular miRNAs, *miR-29b*, *miR-196*, *miR-133a*, *miR-10b* and lower levels of *let-7a*, *let-7b*, *let-7f*, *miR-130a*, and *miR-1a* compared to uninfected CEFs [12]. Cellular miRNAs are also altered in MDV-transformed cell lines compared to non-transformed CD4+ T-cells and include lower expression levels of *miR-155*, *miR-223*, *miR-150*, *miR-451*, *miR-26a*, and *miR-126* [13]. It has been suggested that the downregulation of *miR-26a* is associated with viral transformation, as one of its targets is interleukin 2 (IL2), a regulator of T-cell proliferation [14]. Treatment of MDV-transformed T-cell lines with sodium butyrate, an inducer of viral reactivation, altered the expression of several cellular miRNAs including, *miR-10a*, *miR-130b*, *miR-140-3p*, *miR-2131*, and *miR-3535* [15]. Many of these altered miRNAs likely regulate a number of cellular processes, including DNA replication, DNA damage responses, and cell cycle progression [15]. Taken together, these studies highlight the importance of cellular miRNAs in MDV pathogenesis.

We and others have demonstrated the importance of virally-encoded miRNAs in MDV infections. We also found that co-infection of HVT and MDV alters the expression profile of these miRNAs compared to singular infections. Though several studies have characterized alterations in cellular miRNAs during MDV infections, to our knowledge no studies have been undertaken to determine alterations to the cellular miRNA profile during HVT infections or HVT-MDV co-infections. To further elucidate miRNA regulation during HVT and MDV infections, the current study was undertaken to characterize the impact of HVT and MDV co-infection on the expression of splenic miRNAs.

## 2. Materials and Methods

### 2.1. Birds

All animal procedures were approved by the Institutional Animal Care and Use Committee at North Carolina State University (IACUC No. 10-039-A). Fertilized specific-pathogen-free white leghorn eggs were obtained from Sunrise Farms (Catskills, NY, USA) and incubated using standard conditions. At hatch, birds were divided into four groups. The first group served as unvaccinated, uninfected controls. The second group was vaccinated with HVT (Merial Select, Inc., Gainesville, GA, USA) at one day of age. HVT vaccination was performed according to the manufacturer’s recommended dosage. The third group was vaccinated with HVT at one day of age, then infected with MDV strain MD5 (1000 plaque-forming units) at five days of age. The fourth group was infected with MDV strain MD5 (1000 plaque-forming units) at five days of age. Four chickens from each group were randomly selected at 42 days post MDV infection (dpi), spleens were collected, snap frozen and stored at −80 °C until analyzed.

### 2.2. Small-RNA Sequencing

For small-RNA sequencing (RNA-seq) analysis, three control spleens and three MDV-induced tumorigenic spleens at 42 dpi were used. Total RNA was isolated from 50 mg of splenic tissue using Tri-Reagent (Sigma-Aldrich, St. Louis, MO, USA). RNA was purified following the manufacturer’s instructions with the exception that RNA was precipitated overnight at −20 °C. RNA was quantified using a nanodrop ND-1000 spectrophotometer (Thermo Fisher Scientific, Waltham, MA, USA), and quality of RNA was assessed using a 2100 Bioanalyzer (Agilent Technologies, Santa Clara, CA, USA) with a high sensitivity RNA chip. All RNA samples had RIN values >9. For small RNA-seq library preparation, small RNAs were enriched from total RNA using a miRVana miRNA isolation kit (Ambion, Waltham, MA, USA), and samples were subjected to on-column DNase treatment.

Small RNA libraries for each splenic sample were generated from 1 μg of enriched small RNAs using a TruSeq Small RNA sample preparation kit (Illumina, San Diego, CA, USA) and barcode indices following the manufacturer’s instructions. The quality and quantity of the libraries were assessed on a 2100 Bioanalyzer (Agilent Technologies, Santa Clara, CA, USA) using a high sensitivity DNA chip. Each library was diluted to 10 nM using 10 mM Tris-HCl (pH 8.5) and libraries were pooled. Pooled DNA was sequenced on a single lane of a Genome Analyzer IIx (GAIIx) (Ilumina, San Diego, CA, USA) at the NCSU Genomic Sciences Laboratory.

### 2.3. Data Analysis

All FASTQ sequencing files have been deposited to the Short Read Archive (SRA) database (https://www.ncbi.nlm.nih.gov/sra/) (SAMN10621034-SAMN10621039). All sequencing data processing and analyses were performed using CLC genomics workbench (Qiagen, Germantown, MD, USA). Briefly, FASTQ files were imported into the CLC genomics workbench software. The next generation sequencing trim tool was used to remove any residual adaptor sequences and/or low-quality sequences (Phred < 20). Reads were then mapped to the *Gallus gallus* reference genome (Gallus_gallus-6.0) and normalized using the transformation and normalization tool. Expression analysis of the small RNA libraries was carried out using the small RNA analysis suite.

### 2.4. Yeast One-Hybrid Assays

Yeast one-hybrid analyses were carried out using the Matchmaker Gold yeast one-hybrid system (Clontech, Mountain View, CA, USA) as directed by the manufacturer. The upstream regions (~4 kb) of gga-miR-30d and gga-miR-10b were obtained from Ensembl (http://useast.ensembl.org/index.html), and promoter-like elements were predicted using PROSCAN (https://www-bimas.cit.nih.gov/molbio/proscan/). The regions containing all potential regulatory elements (~500 bp) for each miRNA were individually cloned into the pAbAi vector using KpnI and XhoI and were then sequenced to confirm their identity. Bait strains containing either the *gga-miR-30d* or *gga-miR-10b* promoter cassette were generated following the manufacturer’s instructions. Briefly, Y1H Gold yeast was transformed with 1 µg of either linearized (using BstBI) pAbAi-gga-miR-30d-pro vector or pAbAi-gga-miR-10b-pro vector and yeast with positive cassette integration were selected using SD-Ura media (Clontech). Bait yeast strains were then tested on SD-Ura containing a range of Aureobasidin A (Aba; Clontech) concentrations to determine the optimal Aba concentration for library screening. For the gga-miR30d-pro yeast strain 650 ng/µL Aba was used and for the gga-miR-10b-pro yeast strain 600 ng/µL Aba was used. The complementary DNA (cDNA) library was produced from splenic RNA from four birds for each group; control, HVT, MDV, and HVT + MDV at 42 dpi. For cDNA production, messenger RNA (mRNA) was purified using a NucleoTrap mRNA kit (Clontech). One microgram of mRNA from each sample was pooled, and one microgram of pooled mRNA was used for reverse-transcription using SMART RT (Clontech). SMART cDNA was then used in long-distance PCR to produce a double-stranded cDNA library following the manufacturer’s instructions. Library quality was assessed using gel electrophoresis. The library was purified using a CHROMA SPIN + TE-400 column (Clontech) and concentrated (ethanol/sodium acetate precipitation) as directed by the manufacturer. For each screening, 3 µg of the cDNA library was transformed into the bait yeast following the manufacturer’s instructions and screened on SD-Leu (Clontech) media containing an appropriate amount of Aba. Positive colonies were further selected by re-plating three times. For the *gga-miR-30d* approximately 4.1 million colonies were screened with 42 positive clones (i.e., Aureobasidin A toxin resistant). For the *gga-miR-10b* approximately 3.8 million colonies were screened with 25 positive clones (i.e., Aureobasidin A toxin resistant). Prey clones were sequenced and then mapped to the *Gallus gallus* RefSeq database (https://www.ncbi.nlm.nih.gov/refseq/) to determine their identity.

### 2.5. Real-Time Quantitative Polymerase Chain Reaction

Total RNA was isolated from 50 mg of spleen tissue from four birds in each group, control, HVT, MDV, and HVT + MDV, at 42 dpi using Tri-Reagent (Sigma-Aldrich) following the manufacturer’s instructions with the exception that the RNA was precipitated overnight at −20 °C. Total RNA was DNase-treated using a TURBO-DNA free kit (Thermo Fisher Scientific) following the manufacturer’s instructions. The RNA quality was assessed using agarose electrophoresis. One microgram of DNase-treated total RNA per sample was reverse transcribed using a miScript II RT kit (Qiagen) following the manufacturer’s instructions. For mRNAs, forward and reverse primers were designed using primer-BLAST (https://www.ncbi.nlm.nih.gov/tools/primer-blast/) and for miRNAs, the forward primer consists of the mature miRNA sequence and the reverse primer used was the miScript universal primer (Qiagen). Ribosomal protein L4 (*RPL4*) and the small nucleolar RNA, *snoU83B*, were used as housekeeping genes for mRNA and miRNA normalization, respectively. All primer sequences are provided in Table S1. For small RNA expression analysis, each reaction contained 10 ng of cDNA, 500 nmol of gene-specific forward primer, 1X Universal miScript primer (Qiagen) and 1X iQ SYBR Green Supermix (Bio-Rad, Hercules, CA, USA). The following PCR conditions were used: 95 °C for 5 min, followed by 40 cycles of 95 °C for 10 s, then 58 °C for 20 s. All reactions were performed in duplicate. Gene specific amplification was confirmed using melting curve analysis. The same conditions were also used for gene expression (mRNA) analysis with the exception that 500 nmol of both a gene-specific forward primer and a gene-specific reverse primer was used. Threshold cycle (Ct) values were normalized to the expression levels of *snoU83B* (for miRNAs) or *RPL4* (mRNAs). All expression values are presented relative to the control group. Significant (*p* <0.05) differences in expression were determined using analysis of variance.

## 3. Results

### 3.1. Small RNA-Sequencing Library Characteristics

The total number of high quality (phred >20) mappable reads of the six small RNA libraries (three control spleens; three MDV-induced tumorigenic spleens at 42 dpi) ranged from 1,581,081–4,770,221. The total number of sequenced (Counts Per Million ≥ 20) known chicken miRNA per library ranged from 120–155. The most highly sequenced miRNAs in all libraries were, *miR-148a-3p*, *miR-92-3p*, *let-7f-5p*, *miR-146c-5p*, *let-7a-5p*, *miR-26a-5p*, and *miR-126-3p*. A total of 24 cellular miRNAs were significantly (False Discovery Rate < 0.05) differentially expressed (log_2_ fold-change cutoff of 0.9) between the spleens of control birds and the spleens of MDV-infected birds at 42dpi (Table 1). Of these 13 miRNAs were upregulated: *miR-1458*, *miR-146b-5p*, *miR-146b-3p*, *miR-10a-5p*, *miR-21-5p*, *miR-1b-3p*, *miR-144-3p*, *miR-10b-5p*, *miR-3538*, *miR-146a-5p*, *miR-2188-3p*, *miR-215-5p*, and *miR-147*. The remaining 11 miRNAs were downregulated: *miR-92-3p*, *miR-128-3p*, *miR-218-5p*, *miR-455-5p*, *miR-458a-3p*, *miR-2954*, *miR-1388*, *miR-130b-5p*, *miR-1456-5p*, *miR-30d*, and *miR-122-5p* (Table 1).

### 3.2. MiRNA Real Time-quantitative PCR (RT-qPCR) in the Spleens of Control, MDV-Infected, HVT-Vaccinated, and HVT + MDV-co-Infected Chickens

**Figure 1:**
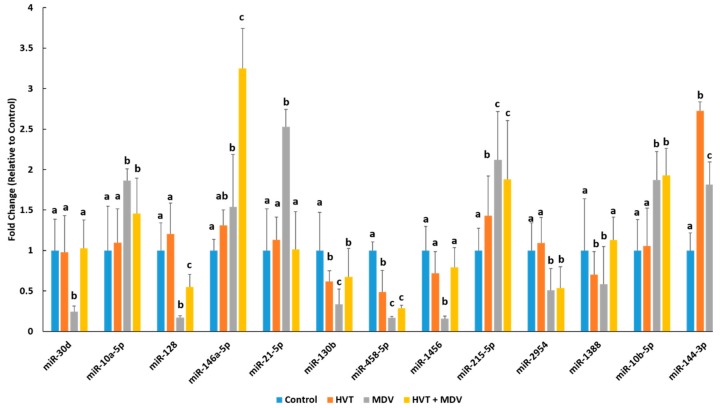
Cellular miRNA splenic expression profiles in uninfected, Herpesvirus of Turkeys (HVT) vaccinated, MDV infected, or HVT + MDV co-infected SPF (specific-pathogen-free) chickens. The splenic expression profile of 13 differentially expressed miRNAs was determined in control (mock-infected) birds (*n* = 4), HVT-vaccinated birds (*n* = 4), MDV-infected birds (*n* = 4), and birds vaccinated with HVT and subsequently infected with MDV (*n* = 4) at 42dpi using RT-qPCR. Error bars denote standard deviations. Differing letters denote statistical significance (*p* < 0.05). For each miRNA, treatment groups with differing letters had a statistically significant difference (*p* < 0.05), while groups with the same letter were not significantly different (*p* > 0.05).

The splenic expression profile of 13 differentially expressed miRNAs were determined in control birds (*n* = 4), HVT-vaccinated birds (*n* = 4), MDV-infected birds (*n* = 4), and HVT + MDV co-infected birds (*n* = 4) at 42 dpi using RT-qPCR (Figure 1). In the small RNA-seq experiment, *miR-30d* was expressed 3.78-fold (log_2_ 1.92) lower in the spleens of MDV-infected birds (Table 1). This is in good agreement with the RT-qPCR analysis, which found a 4.17-fold reduction of *miR-30d* expression in the spleens of MDV-infected birds (Figure 1). Interestingly, the expression of *miR-30d* was significantly repressed in MDV-infected birds but was not different between control, HVT-vaccinated, or HVT + MDV co-infected birds (Figure 1). Small RNA-seq analysis found that *miR-10a-5p* was 2.8-fold (log_2_ 1.51) higher in the spleens of MDV-infected birds than in the spleens of control birds. The RT-qPCR analysis confirmed this upregulation of *miR-10a-5p* in MDV-infected birds (Figure 1). *MiR-10a-5p* was also significantly increased in HVT + MDV co-infected birds, but HVT-vaccinated birds did not have a significantly different *miR-10a-5p* expression from control birds. The closely related miRNA, *miR-10b-5p* also had significantly higher expression in the spleens of MDV-infected birds and HVT + MDV co-infected birds compared to their control and HVT-only counterparts (Table 1; Figure 1). Both small RNA-seq and RT-qPCR analyses found an increase in *miR-146a-5p* expression in the spleens of birds infected with MDV compared to uninfected birds (Table 1; Figure 1). *MiR-146a-5p* was significantly higher in the spleens of HVT + MDV co-infected birds (3.25 fold) compared to birds in the other three groups. The miRNA *miR-21-5p* was significantly increased in the spleens of MDV-infected birds (Table 1 and Figure 1) but was not significantly different between the other three groups (Figure 1). The miRNA *miR-144-3p* was similarly upregulated in birds singularly infected with HVT or HVT + MDV co-infected birds (Figure 1). It was significantly higher in MDV-infected birds, but to a lesser extent (Figure 1).

### 3.3. Yeast One-Hybrid Screenings of the Chicken miR-30d Promoter and the miR-10b Promoter

**Figure 2:**
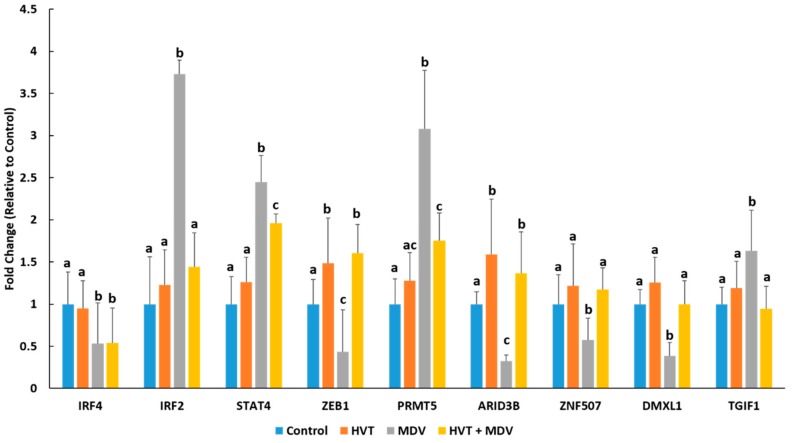
The splenic expression profile transcriptional regulators identified in yeast one-hybrid screenings of the chicken promoters of *miR-30d* and *miR-10b*. Expression was determined in control (mock-infected) birds (*n* = 4), HVT-vaccinated birds (*n* = 4), MDV-infected birds (*n* = 4), and birds vaccinated with HVT and subsequently infected with MDV (*n* = 4) at 42 dpi using RT-qPCR. Error bars denote standard deviations. Differing letters denote statistical significance *(p* < 0.05). For each gene, treatment groups with differing letters had a statistically significant difference (*p* < 0.05), while groups with the same letter were not significantly different (*p* > 0.05).

A yeast one-hybrid screening of the *miR-30d* promoter identified five potential regulatory proteins, IRF2, ARID3B, DMXL1, ZNF507, and TGIF1 (Table 2). Gene Ontologies and predicted functional partners for each identified transcription factor are also provided in Table 2. RT-qPCR analysis revealed that *IRF2* is significantly higher in the spleens of MDV-infected birds, but not in HVT-vaccinated or HVT + MDV co-infected birds relative to control birds (Figure 2). *ARID3B* was significantly lower in MDV-infected birds compared to the other groups, as was DMXL1 and ZNF507 expression (Figure 2). *TGIF1* expression was only significantly different in MDV-infected birds (Figure 2). In the yeast one-hybrid screening of the *miR-10b* promoter four potential regulatory proteins were discovered, IRF4, PRMT5, ZEB1, and STAT4 (Table 2). *IRF4* was significantly reduced in the spleens of MDV-infected and HVT + MDV co-infected birds relative to control and HVT-vaccinated birds (Figure 2). *PRMT5* was significantly induced in MDV-infected and to a lesser extent in HVT + MDV co-infected birds compared to the other two groups. (Figure 2). *ZEB1* expression was significantly lower in MDV-infected birds relative to the other groups (Figure 2). It was higher in HVT vaccinated and HVT-MDV co-infected birds compared to control birds (Figure 2). *STAT4* expression was lower in control and HVT-vaccinated birds than in MDV-infected or co-infected birds (Figure 2).

## 4. Discussion

In the present study, we discovered 24 cellular miRNAs, which were altered in the spleens of MDV-infected specific-pathogen-free (SPF) white leghorns at 42 dpi using small RNA-seq. We chose to use 42 dpi as this is considered as the transformation (lymphoma development) stage of MD pathogenesis [16]. We subsequently profiled the splenic expression of a select group of these miRNAs in birds vaccinated with HVT or birds which were HVT-vaccinated and subsequently infected with MDV. Many of these miRNAs were uniquely altered in the spleens of vaccinated/infected birds versus spleens from uninfected birds or birds singularly infected with either HVT or MDV. To further elucidate miRNA-mediated regulatory mechanisms in MDV infection we employed a yeast one-hybrid system to identify potential transcriptional regulators of two differentially expressed miRNAs. We found *miR-30d*, which has decreased splenic expression in MDV-infected birds, is potentially regulated by IRF2, ARID3B, DMXL1, ZNF507, and TGIF1. The expression of an upregulated miRNA *miR-10b-5p*, is likely, at least in part, regulated by IRF4, PRMT5, ZEB1, and STAT4. Taken together, these results suggest a complex transcriptional and post-transcriptional regulatory system contributes to both protection from and enhancement of disease progression in gallid herpes viral infections. 

Here, we found that the cellular miRNA, *miR-144-3p* was similarly significantly upregulated in the spleens of both HVT vaccinated birds and vaccinated birds subsequently infected with MDV (Figure 1). Even though birds infected with MDV had higher *miR-144-3p* splenic levels than uninfected birds, this increase was more subdued than in two groups of birds receiving the HVT vaccine (Figure 1). Several studies have shown in human lung cancer malignancies *miR-144-3p* is downregulated [17,18]. Furthermore, *miR-144-3p* was found to target several known oncogenes, including SLC44A5, MARCKS, and NCS1 [17]. Ectopic *miR-144-3p* expression in lung cancer cells reduced cell proliferation, migration, and invasion [17,18]. Our finding that *miR-144-3p* splenic expression was significantly higher in MDV-infected birds that received HVT vaccination versus infected birds that did not receive the vaccine, suggests that *miR-144-3p* may contribute to HVT-mediated protection against virally-induced cellular transformation. 

The cellular miRNA *miR-128* displayed similar splenic expression between control birds and HVT vaccinated birds (Figure 1). Its expression was severely repressed in birds infected with MDV. While this expression was more muted in HVT vaccinated birds subsequently infected with MDV compared to control birds or those solely receiving the vaccine, it was still significantly higher than MDV-infected birds not receiving the vaccine (Figure 1). *MiR-128* is known to promote apoptosis and reduce tumor cell growth and invasiveness [19,20]. This reduction is likely due, in part, to a reduction in COX2, which is often upregulated in transformed cells [19]. Here, we found that HVT induces much higher splenic levels of *miR-128* than MDV and that prior HVT vaccination also significantly increases *miR-128* levels in MDV-infected birds (Figure 1). Therefore, it is possible that *miR-128* may contribute to protection from cellular transformation and tumor progression in HVT vaccinated chickens that are subsequently infected with an oncogenic strain of MDV.

We found that *miR-21-5p* splenic expression was highly induced by MDV infection (Figure 1). However, birds solely vaccinated with HVT and birds co-infected with HVT and MDV had comparable *miR-21-5p* levels to uninfected birds (Figure 1). *MiR-21-5p* is a well-known oncomiR [20]. It is known to be highly expressed in a myriad of cancers [21,22]. It is also induced by inflammatory responses in hematopoietic cells [21]. *MiR-21-5p* overexpression has recently been linked to EBV transformation of B-cells [23]. Taken together, these studies suggest that very high *miR-21-5p* levels found here in the spleens of MDV-infected birds compared to HVT vaccinated birds suggests that it may be a contributing factor to MDV transformation of infected T-cells. It is possible HVT mutes *miR-21-5p* expression through differential inflammatory responses than MDV, though further work is necessary in order to confirm this.

The immunomodulatory miRNA *miR-146a-5p* regulates a number of pathways in immune cells [24]. We found that splenic levels of *miR-146a-5p* were much higher in chickens receiving the HVT prior to MDV infection than chickens singularly infected with either virus (Figure 1). Out of all hematopoietic cell types *miR-146a* is most highly expressed in CD4+ helper T-cells and germinal center B-cells [24]. One of its functions is to mute T-cell activation and prevent excessive T-cell numbers [24]. As MDV transforms activated CD4+ T-cells [25], it is possible that the much higher levels of *miR-146a-5p* in MDV birds receiving the vaccine versus infected birds not receiving the vaccine may reduce the number of available permissive T-cells and may, therefore, limit MD progression.

We found that *miR-30d* expression is significantly reduced in the spleens of MDV-infected birds but had comparable expression between uninfected, HVT vaccinated and HVT + MDV co-infected birds (Figure 1). In human cancers, *miR-30d* expression is often downregulated [26,27]. Transfection of a *miR-30d* mimic into colon cancer cells inhibited autophagy and induced apoptosis [27]. Ectopic *miR-30d* expression also led to cell cycle arrest [27]. These studies suggest *miR-30d* is an important mediator of cell growth processes and may be linked to neoplasia of MDV-infected cells. To further discern the involvement of *miR-30d* in HVT protection and MDV pathogenesis we used a yeast one-hybrid screen to discover potential regulators of *miR-30d* in chickens. This screen identified five proteins which potentially interact with the chicken *miR-30d* promoter, IRF2, ARID3B, DMXL1, ZNF507, and TGIF1. ARID3B is a member of the ARID (AT-rich domain) transcription factor family, which play a number of roles in cell fate and the cell cycle [28]. ARID3B was recently linked to lytic reactivation of KSHV [29]. In KSHV-infected cells, the viral protein RTA, the major KSHV latent/lytic switch was found to induce ARID3B expression [29]. Reduction of ARID3B expression induced KSHV reactivation, while overexpression of ARID3B blocked reactivation [29]. If a similar mechanism exists in MDV, then MDV-induced alterations in ARID3B expression (Figure 2) impacts *miR-30d* in infected cells, ultimately leading to dysregulation of cell proliferation processes. IRF2 is transcriptional repressor induced by INF1α/β as part of an interferon signaling feedback loop [30]. We found that IRF2 is much higher in MDV-infected birds than in their uninfected or HVT vaccinated counterparts (Figure 2). This could, at least in part, explain the reduction of *miR-30d* in MDV-infected birds. In EBV-transformed B-cells, overexpression of IRF2 is associated with cellular transformation, while IRF2 knockout in these cells leads to apoptosis [31]. IRF2 also repressed type III latency in EBV-infected cells by repressing the activity of the viral BamHI Q promoter [32]. It is possible that increased IRF2 levels in MDV-infected birds result in decreased *miR-30d* levels, contributing to increased cell proliferation rates, as has been found with IRF2 functions in other herpes viral infections.

The miRNA, *miR-10b-5p* was significantly higher in the spleens of MDV infected chickens compared to the other three groups (Figure 1). This miRNA is upregulated in a number of human cancers and its oncogenic potential is associated with its ability to regulate *HOX* genes and Kruppel-like factors [33]. Using a yeast one-hybrid analysis, we identified four potential transcriptional factors which may regulate *miR-10b* expression in chickens, IRF4, PRMT5, ZEB1, and STAT4. IRF4 is a hematopoietic cell-specific transcriptional regulator, which is induced in response to type I interferons [34]. IRF4 induces the expression of a subset of IFN-stimulated genes in response to interferon signaling [34]. One of these genes is *CIITA*, an activator of Major Histocompatibility Complex II (MHCII) expression, to ultimately regulate T-cell activation [35]. The KSHV latency transcript LANA prevents IRF4 activation of the CIITA promoter by physically interacting with IRF4 to prevent it from binding the promoter [34], suggesting an important role for IRF4 signaling in the adaptive immune response to herpes viral infections. This is supported by the fact that KSHV encodes its own version of IRF4 to block cellular IRF4 function, which it uses to suppress host cell antiviral immune responses and to circumvent host cell growth regulation [36]. Induction of IRF4 expression as part of the cellular antiviral response to MDV may lead to increased *miR-10b-5p*, which inadvertently may contribute to viral pathogenesis. We found *ZEB1* expression was significantly reduced in the spleens of MDV infected birds (Figure 2). ZEB1, a zinc finger protein, is a transcriptional repressor of IL2 and BCL6 [37]. ZEB1 expression closely linked with the lytic/latent switch in several herpesviruses, including EBV [38,39] and HSV1 [40]. ZEB1 can repress the expression of the *miR-183* family of miRNAs in humans [40]. THE HSV1 encoded ICP0 ubiquitin ligase actively degrades ZEB1 to increase the production of the *miR-183* family which is needed for a productive HSV1 infection [40]. Repression of ZEB1 expression by the cellular miR-200 family has been linked to lytic reactivation of EBV [9]. Interestingly, *miR-10b-5p* can also target ZEB1 [41]. This suggests that MDV and possibly other herpesviruses induce a ZEB1/*miR-10b* negative feedback loop to create a more favorable environment for a productive infection.

In the present study, RT-qPCR analysis of the splenic expression of the MDV oncogene, *MEQ*, indicates that HVT vaccination greatly reduced MDV replication compared to the MDV-infected birds not receiving the HVT vaccine (Figure S1). However, RT-qPCR of the HVT pp38 homolog, HVT071, indicates that HVT replication was comparable between birds only receiving the HVT vaccine and HVT vaccinated birds subsequently infected with MDV (Figure S1). In general, the splenic expression of the miRNAs and their transcriptional factors profiled here, was more divergent in MDV-infected birds, compared to birds either singularly infected with HVT and those co-infected with HVT + MDV (Figure 1; Figure 2). It is interesting to speculate that alterations in cellular miRNA-mediated regulation of host immune processes and possibly viral gene expression, as discussed above, may contribute the reduced replicative ability of MDV in HVT vaccinated birds, however future studies pinpointing specific miRNA functions are needed to confirm this.

## 5. Conclusions

In conclusion, we have found that a subsect of host miRNAs has unique splenic expression profiles between chickens infected with HVT, MDV, or co-infected with HVT and MDV. We also discovered a group of transcription factors that regulate immune responses and cell growth pathways and are also likely to regulate the expression of miRNAs. Overall, the results presented here suggest that cellular miRNA-mediated post-transcriptional gene regulation is a critical component of both protection from and progression of Marek’s Disease.

## Figures and Tables

**Table 1:** Cellular micro RNAs (miRNAs) differentially expressed (minimum CPM cutoff of 20; log_2_ fold change cutoff of 0.9; and FDR < 0.05) between mock-infected and Marek’s Disease Virus (MDV)-infected spleens at 42 dpi identified by small RNA-sequencing.

miRNA	Log_2_ Fold-Change
*miR-1458*	1.68
*miR-146b-5p*	1.65
*miR-146b-3p*	1.57
*miR-10a-5p*	1.51
*miR-21-5p*	1.48
*miR-1b-3p*	1.30
*miR-144-3p*	1.26
*miR-10b-5p*	1.14
*miR-3538*	1.14
*miR-146a-5p*	1.08
*miR-2188-3p*	1.02
*mir-215-5p*	1.00
*miR-147*	0.92
*miR-92-3p*	−0.92
*miR-128*	−0.93
*miR-218-5p*	−1.02
*miR-455-5p*	−1.04
*miR-458a-3p*	−1.12
*miR-2954*	−1.16
*miR-1388*	−1.20
*miR-130b-5p*	−1.22
*miR-1456-5p*	−1.23
*miR-30d*	−1.92
*miR-122-5p*	−2.12

**Table 2:** Transcriptional factors identified in yeast one-hybrid screening of the chicken *miR-30d* and *miR-10b* promoters.

**Transcription Factor**	**Gene Ontology: Biological Process ^1^**	**Predicted Functional Partners ^2^**
***miR-30d***		
IRF2	negative regulation of transcription by RNA polymerase II; transcription, DNA-templated; regulation of transcription, DNA-templated; transcription by RNA polymerase	IRF1; IRF7, IRF4, GTF2B; KAT2B; REL; HMGB1; NR2C1; KAT2A; IRF6
ARID3B	transcription, DNA-templated; regulation of transcription, DNA-templated; transcription by RNA polymerase II; positive regulation of transcription by RNA polymerase II	SMARCD1; SMARCD2; SMARCD3; MYSM1; UBL7; HSP90AB1; ACTL6A
DMXL1	regulation of transcription, DNA-templated; transcription by RNA; transcription by RNA polymerase II; vacuolar acidification	ATP6V1B2; ATP6V1A; ATP6V1C1; ATP6V1C2; ATP6V1E1; PBRM1; SKP1; PIKFYVE
ZNF507	transcription, DNA-templated; regulation of transcription, DNA-templated; regulation of transcription by RNA polymerase II	RIPK4; DNAJC11; WDR90; CDYL2; CEP57; MPHOSPH8; ACTR10L; ACTR10
TGIF1	RNA polymerase II proximal promoter sequence-specific DNA binding; RNA polymerase II transcription factor activity, sequence-specific DNA binding; DNA binding transcription factor activity	TGIF2; MADH2; SMAD2; SMAD3; SIN3A; SIN3B; PBX1; PBX3; PBX4; HDAC2
***miR-10a***		
IRF4	transcription, DNA-templated; regulation of transcription, DNA-templated; transcription by RNA polymerase II; protein methylation; peptidyl-lysine methylation	BCL6; PRDM1; IL4R; CD40LG; SPI1; BATF; IL2RG; IRF1; IRF2; REL
PRMT5	spliceosomal snRNP assembly; chromatin organization; transcription, DNA-templated; DNA-templated transcription, termination; regulation of transcription, DNA-templated	CLNS1A; GEMIN2; GEMIN4; GEMIN6; CDC25A; CDC25B; CDK6; SNRPD1
ZEB1	transcription, DNA-templated; regulation of transcription, DNA-templated; regulation of transcription by RNA polymerase II; immune response	SBDS; ASL1; VIM; CDH1
STAT4	transcription, DNA-templated; regulation of transcription, DNA-templated; signal transduction; JAK-STAT cascade; cytokine-mediated signaling pathway	JAK1; JAK2; TYK2; IFNAR1; PIAS1; PIAS2; PIAS4; CISH; EP300; CREBBP

^1^ Gene Ontology Biological Process categories obtained from http://www.geneontology.org; ^2^ Predicted Functional Partners obtained from https://string-db.org.

## Supplementary Materials

The following are available online at http://www.mdpi.com/2073-4425/10/2/115/s1 Table S1: Primers used in this study, Figure S1: Splenic expression of MDV *MEQ* and HVT *HVT071* in HVT-vaccinated, MDV-infected or HVT + MDV co-infected SPF chickens.
