# Supplementary material for: Impact of HVT Vaccination on Splenic miRNA Expression in Marek’s Disease Virus Infections
Source: Genes (Basel). 2019 Feb 5;10(2):115. doi: 10.3390/genes10020115 (PMC6409792; doi:10.3390/genes10020115)
Supplement: Supplementary file 1 [file genes-10-00115-s001.zip › Figure S1.pdf]

Figure S1. Analysis of viral gene expression.

To assess infection status of the birds used in this study RT-qPCR analysis was used to assay the expression levels of the MDV-oncogene, MEQ, and the HVT pp38 homolog, HVT071. The MEQ sequence was obtained from the MDV strain MD5 complete genome sequence (genbank id: NC_002229.3). The HVT071 sequence was obtained from the HVT strain FC126 whole genome sequence (genbank id: AF291866.1). Primers were designed using primer-BLAST (https://www.ncbi.nlm.nih.gov/tools/primer-blast/). During the design, MEQ primers were crosschecked with the Gallus gallus and HVT FC126 genomes to ensure specificity, likewise HVT071 primers were crosschecked with the Gallus gallus and MDV MD5 genomes to ensure their specificity. Primer sequences are provided in Table S1. RT-qPCR analysis was performed as follows. Each reaction contained 10 ng of cDNA 500 nmol each of the forward and reverse primers and 1X iQSYBR Green Supermix (Bio-Rad). All reactions were performed in duplicate. The following PCR conditions were used: 95°C for 5 minutes, followed by 40 cycles of 95°C for 10 seconds, then 58°C for 20 seconds. Gene specific amplification was confirmed using melting curve analysis. Threshold cycle (Ct) values were normalized to expression levels of RPL4. Significant ($p<0.05$) differences in expression were determined using analysis of variance.

Both MEQ and HVT071 expression was assessed in all four birds in all four treatment groups used in this study. Neither viral gene could be detected in the spleens of uninfected control birds. MEQ could not be detected in birds only receiving the HVT vaccine, likewise HVT071 could not be detected in birds singularly infected with MDV. Therefore, these comparisons are not included in the graph. MEQ splenic expression was much higher (log_2 9.03; $p<0.001$) in birds singularly infected MDV compared to MDV-infected birds receiving the HVT vaccine at 42dpi (Figure S1). HVT071 was similarly expressed in the spleens of birds receiving HVT vaccine only and HVT-vaccinated birds subsequently infected with MDV at 42 dpi (Figure S1). There was slightly higher HVT071 expression in co-infected birds, but this increase was not statistically significant ($p>0.05$). In general, HVT071 was lower expressed than MEQ. Overall, these results suggest that HVT vaccination reduced MDV replication.

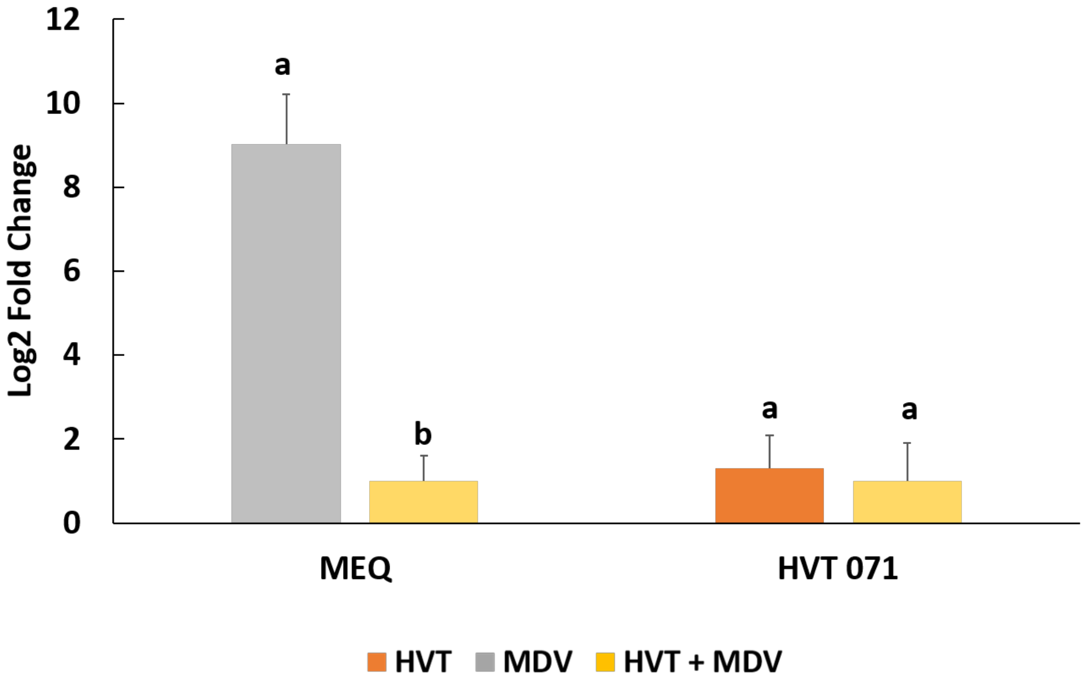

Figure S1. Splenic expression of MDV MEQ and HVT HVT071 in HVT-vaccinated, MDV-infected or HVT + MDV co-infected SPF chickens. Expression was determined in control (mock-infected) birds (n=4), HVT-vaccinated birds (n=4), MDV-infected birds (n=4), and birds vaccinated with HVT and subsequently infected with MDV (n=4) at 42dpi using RT-qPCR. Error bars denote standard deviations. Differing letters denote statistical significance ($p<0.05$). For each gene, treatment groups with differing letters had a statistically significant difference ($p<0.05$), while groups sharing a letter were not significantly different. NOTE: Neither viral gene could be detected in the spleens of uninfected control birds. MEQ could not be detected in birds only receiving the HVT vaccine, likewise HVT071 could not be detected in birds singularly infected with MDV. Therefore, these comparisons are not included in the graph.
